# Analytical study of ^226^Ra activity concentration in market consuming foodstuffs of Ramsar, Iran

**DOI:** 10.1186/s40201-017-0281-3

**Published:** 2017-09-07

**Authors:** M. Gooniband Shooshtari, M. R. Deevband, M. R. Kardan, N. Fathabadi, A. A. Salehi, K. Naddafi, M. Yunesian, R. Nabizadeh Nodehi, M. Karimi, S. S. Hosseini

**Affiliations:** 1 Department of Medical Physics and Biomedical Engineering, School of Medicin, Shahid Beheshti University of Medical Sciences, Tehran, Iran; 2 Nuclear Science and Technology Research Institute, Tehran, Iran; 3 Center for Air Pollution Research (CAPR), Institute for Environmental Research (IER)Tehran University of Medical Sciences, Tehran, Iran; 4 Environmental Radiological Protection Division, National Radiation Protection Department, Iran Nuclear Regulatory Authority, Atomic Energy Organization of Iran (AEOI), Tehran, Iran; 5 Department of Energy Engineering, Sharif University of Technology, Tehran, Iran; 6 Department of Environmental Health Engineering, School of Public Health, Tehran University of Medical Sciences, Tehran, Iran

**Keywords:** Market foodstuff, Radioactivity, ^226^Ra, Ramsar, α-spectrometry

## Abstract

**Background:**

Ramsar, a city of Iran located on the coast of the Caspian Sea, has been considered to be enormously important due to its high natural radioactivity levels. People living in High Level Natural Radiation Areas (HLNRAs) have been exposed by several sources, one of which could be foodstuff. However, many studies have been carried out to measure the environmental radioactivity in Ramsar, but no survey has been conducted in all stapled consumed foods yet. This study was dedicated to determine ^226^Ra activity concentration in the daily diets of Ramsar residents as a probable exposure.

**Methods:**

Approximately 70 different market samples were collected during the four seasons based on the daily consumption patterns of residents which have the highest consumption and their availability in the seasons. All samples, after washing, drying and pretreatment, were analyzed for ^226^Ra radionuclide determination by α-spectrometry.

**Results:**

The mean radioactivity concentration of ^226^Ra ranged between 7 ± 1 mBq Kg^−1^ wet weight in meat, and 318 ± 118 mBq Kg^−1^ for tea dry leaves. The ^226^Ra activity concentrations in collected samples varied from below the minimum detectable activity up to 530 ± 30 mBq Kg^−1^. To compare the results with United Nations Scientific Committee on Effects of Atomic Radiation (UNSCEAR) reference values, the ^226^Ra activity concentrations concluded from the results appear to be higher in milk, chicken and eggs and less in grain products, vegetables, fruits and fish products. These results indicate that no significant ^226^Ra contamination is present in market foodstuffs and provide reference values for the foodstuffs in Ramsar.

**Conclusions:**

Of the total daily dietary ^226^Ra exposure from market consuming foodstuffs for adults in Ramsar, the largest percentage was from wheat. The residents consuming wheat and manufacturing wheat products such as bread, pasta, porridge, crackers, biscuits, pancakes, pies, pastries, cakes, cookies, muffins, rolls, doughnuts, breakfast cereals and so on may receive an elevated dose in the diet. In conclusion, with regards to presence of ^226^Ra in foodstuffs it is necessary to monitor regularly the activity of ^226^Ra in foodstuffs including market and local foods.

## Background

In most areas in the world, the natural radioactivity varies only within a certain limit, while there are a few regions that are known to have high background radiation areas. These are due to the local geological properties and geochemical effects that cause increased levels of terrestrial radiation [1]. A very high background radiation area was found at Ramsar in Iran, a type of area known as a High Level Natural Radiation Area (HLNRA) [2].

The major contribution to radiation exposure comes from natural radionuclides of both terrestrial and cosmogenic origins, which have caused approximately 85% of the annual total dose of the population [3]. Among natural radionuclides, the α-emitters are significant due to their potential internal human radiation exposure [4]. As radium chemical behavior is similar to that of calcium in the body, and radium has been known to be one of the most radiotoxic radionuclides, this radionuclide was therefore chosen to be determined in market food samples [5].

Natural and artificial radioactivity measurements have been carried out in foodstuffs [6–10]. Also, a number of studies have been carried out on market foodstuffs [11–15]. The United Nations Scientific Committee on the Effects of Atomic Radiation’s report is the relevant literature that contains major reviews on the radioactivity of foodstuff [3]. Different radiological measurements and epidemiological studies have been carried out on Ramsar in Iran, due its importance as an HLNRA [1, 16]. Few investigators have measuring the radioactivity of foodstuff in Iran [5, 17, 18]. Among these literatures, a study has been carried out to experimentally determine the vegetable-to-soil concentration ratio (CR) of ^226^Ra in HLNRAs of Talesh Mahalleh in Ramsar. An estimation of the effective dose due to the ingestion of only edible vegetables, rather than all the daily diet, has been taken from a critical group rather than all the residents in this region [19]. Another study has been conducted to set a proper method for measuring the low concentration of ^226^Ra and ^224^Ra in fruit and vegetable samples taken from Ramsar [4].

As consumption of food is usually one of the most important routes by which natural and artificial radionuclides can intake by the human body, an evaluation of radionuclide levels in different food samples and residents’ diets is very important in order to determine this [20].

As far as Ramsar is concerned, there are few data that show the different major food groups that are consumed the most by residents, so this study was performed in order to determine the ^226^Ra concentration in foodstuff obtained from the Ramsar markets. In most cities, the foodstuff consumed by residents includes both local and marketing products. This is also the case in Ramsar, so market products would be one of the ways that people are exposed to ^226^Ra in food. Among the different kinds of measurement methods for ^226^Ra determination, alpha-spectrometry has been chosen because of its lower detection limit [4].

Food and Agriculture Organization (FAO) and International Atomic Energy Agency (IAEA) have established guideline levels for the artificial radioactive substances in foodstuffs moving in international trade [21, 22], but there are no guidelines for local foodstuffs.

The purposes of this study were to present ^226^Ra concentrations in different major market samples in the daily diet of residents’ foods within the city of Ramsar. Information on the levels of naturally occurring radionuclide is important as they contribute to a substantial fraction of the radiation dose to the natural ecosystems; therefore, the identification of information relevant to exposures in this area is another key factor in transfer pathway to people and have noticeable portion on public health.

## Methods

**Fig. 1:**
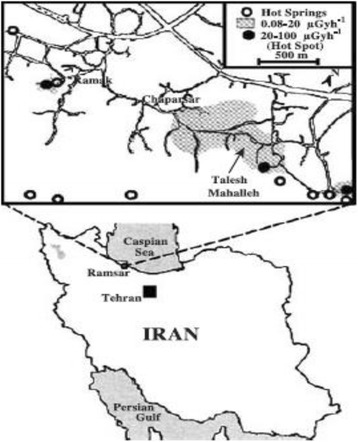
The geographical location and radiation map of Ramsar [19]

Ramsar is a coastal city in the province of Mazandaran, Iran, located on the west side of Caspian Sea; Ramsar is known to have a high background radiation region. The geographical location and radiation map of Ramsar is shown in Fig. 1. [19].

### Foodstuff samples collection and preparation

Different food samples were collected from Ramsar markets. These selected samples were the quintessence of the samples in the diet of residents according to the data gathered from the National Nutrition and Food Technology Research Institute, which included the most consumed foodstuff of each province [23]. Seventy different market food staples, which included major food groups, were selected and analyzed by the alpha-spectrometry method. The fresh samples were collected during the period of this study according to seasonal availability. A number of selected samples from each type of foodstuff were based on the amounts of consumption and their contribution to the daily diet of the population. The sampling was carried out during four different seasons (2015–2016) in order to take into account different staples of foodstuff, such as citrus fruits, which are only available in one season.

These samples were obtained from major market distribution centers which covered different products in Ramsar city.

It should be mentioned that the aim of this study was to include different market samples according to the eating habits of the people living in the regions and prepared from different foodstuff centers. The samples of this study area included six major food groups: 1) Meat products, including meat, chicken and eggs; 2) Milk products, including milk and cheese; 3) Grain products, including rice and wheat; 4) Vegetables, including basil, mint, coriander, parsley, radish leaves; 5) Fruit, which included only citrus, because the citrus is the main fruit production of this area and 6) fish products included different types of fish.

In Ramsar, the diets of inhabitants include crops both locally grown and raised and imported from other areas. Some of the foods such as rice, duck eggs and fish were gotten from the farmers, local producers, and so on (“local”), while the others of foodstuffs such as meat, chicken and wheat were mostly bought in the grocer’s shops, supermarkets and so on (“market”) where they are sourced from the whole of the country. Other foodstuffs represent the food basket of Ramsar population were provided with different percentages from both sources. In this study we focused on market foodstuffs that are sourced from the whole of the country and supplied in the Ramsar’s markets. Numbers of selected samples from each kind of foodstuff were based on the amounts of consumption and their contribution to the daily diet of the people. To prepare the samples, first they were washed and the non-edible parts were removed and weighed for determination of their fresh weight. Then, the samples were turned to ash for about 16 h at 300 °C in the first oven, and then turned to ash again in the next oven at 700 °C for 16 h [7].The six samples were analyzed for each kind of foods. Table 1 presents the ratio between the ash and wet weights in the selected samples.

**Table 1:** The ratio of ash weight to wet weight of foodstuff staples of Ramsar

Type of foodstuff	W_ash_/W_wet_(gr)
Meat	0.031
Chicken	0.084
Fish	0.032
Egg	0.007
Rice	0.004
Milk	0.089
Cheese	0.053
Vegetables	0.014
Tea	0.063
Citrus	0.003
Wheat	0.057

### Radioactivity measurement

There are several techniques for the identification and measurement of radionuclides, such as α-spectrometry, liquid scintillation procedure counting (LSC), solid state nuclear track detector, and γ-ray spectrometry, which is based on their gain and loss [4, 24–26].

A quality assurance system according to International organization for standardization (ISO-17025) requirements in these analyses has been implemented as an important factor for producing reliable and valid analytical results, as well as an estimation of measurement results.

Three grams of the each ash of sample was weighted in a 100 ml beaker and then ^133^Ba tracer solution was used to determine the radium recovery factor. Leaching of the samples was done by HNO_3_ solution and was then continued by HClO_4_ solution as necessary. Leaching was finalized with HNO_3_ and H_2_O_2_ solutions. Radium was precipitated as a form of sulfate. The precipitate was purified by dissolving in an Ethylene diamine triacetic acid (EDTA) solution and then repeating the precipitation procedure. Finally, the micro-precipitate of Ba(Ra) SO_4_ was prepared by adding a small amount of barium carrier [5]. A validation method was performed by analyzing the reference materials prepared from the International Atomic Energy Agency [22]. The radium recovery factor was determined by a gamma spectrometry system, using a high-purity germanium (HPGe) detector with 40% relative efficiency through ^133^Ba photo peak (356 keV). Measurement of ^226^Ra was performed by alpha-spectrometry with a silicon surface barrier detector connected to the Multi-channel analyzer (MCA) and vacuum chamber. The sample’s activity concentration was then calculated in mBq/kg in ash weight. The minimum detectable activity (MDA) is 1mBq in our geometry and the counting time was 60,000 s. It needs to be mentioned that the activity concentration measured in ash weight were converted back to wet weight using data presented in Table 1.

## Results

The 70 samples, which represented the market produce and had the highest consumption rates, were collected and analyzed based on the above-mentioned method for the determination of ^226^Ra concentration. Table 2 shows the ^226^Ra mean activity concentrations and related standard deviations found in various foodstuffs. The mean activity concentration in the meat and egg samples ranged from 7 ± 1 (mBq/kg wet weight) to 48 ± 9 (mBq/kg wet weight) respectively. In this group as results showed, the maximum activity concentration of ^226^Ra was found in egg by the amount of 65 ± 6 (mBq/kg wet weight) and minimum activity concentrations in meat that was below the minimum detectable activity of the system(< MDA).

**Table 2:** The minimum, maximum and mean activity concentration of ^226^Ra (mBq/kg wet weight) in foodstuff staples of Ramsar (mean ± SD; *n* = 6)

Major group of foodstuff	Type of foodstuff	Mean activity ± SD (mBq/Kg)	Min activity (mBq/Kg)	Max activity (mBq/Kg)
Meat	Meat	7 ± 1	MDA	9 ± 3
	Chicken	27 ± 15	7 ± 3	57 ± 6
	Egg	48 ± 9	38 ± 4	65 ± 6
Grain	Wheat	51 ± 14	32 ± 3	70 ± 7
	Rice	41 ± 10	28 ± 4	54 ± 6
Milk	Milk	39 ± 23	4 ± 1	64 ± 18
	Cheese	43 ± 5	38 ± 4	51 ± 6
Vegetables	vegetables	30 ± 11	10 ± 3	45 ± 8
	Tea	318 ± 118	163 ± 20	530 ± 30
Fruit	Citrus	10 ± 4	7 ± 1	16 ± 3
Fish	Fish	32 ± 8	18 ± 3	43 ± 7

The mean activity of ^226^Ra in grain products were found from 41 ± 10 (mBq/kg wet weight) to 51 ± 14 (mBq/kg wet weight). The maximum and minimum activity concentration of ^226^Ra in this group were 70 ± 7 (mBq/kg wet weight) in wheat and 28 ± 4 (mBq/kg wet weight) in rice.

The mean activity concentration of ^226^Ra in milk products ranged between 39 ± 23 (mBq/kg wet weight) to 43 ± 5 (mBq/kg wet weight) in milk and cheese samples. The results represented that the maximum and minimum activity concentration of ^226^Ra in milk were 64 ± 18 (mBq/kg wet weight) and 4 ± 1 (mBq/kg wet weight). For vegetables, this mean scale was 30 ± 11 (mBq/kg wet weight) which the minimum and maximum activity concentration were 10 ± 3 (mBq/kg wet weight) and 45 ± 8 (mBq/kg wet weight).

The average activity concentration in citrus found 10 ± 4 (mBq/kg wet weight) by minimum and maximum amount of 7 ± 1(mBq/kg wet weight) to 16 ± 3 (mBq/kg wet weight).

**Fig. 2:**
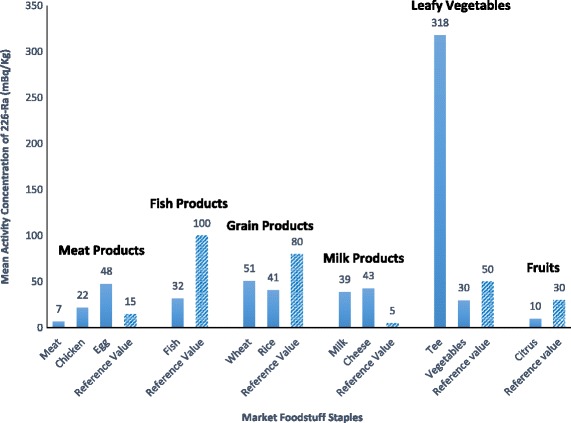
Mean activity concentration of ^226^Ra(mBq/kg) in market foodstuff staples of Ramsar

As shown on Fig. 2, the mean radioactivity concentration of ^226^Ra in all samples ranged between 7 ± 1 (mBq/kg wet weight) that related to meat and 318 ± 118 mBq Kg-1 for tea dry leaves. These results indicate that the ^226^Ra level in tea is higher than the other market foodstuffs found in Ramsar, with a minimum activity concentration found in meat, which is below the MDA. This difference in concentrations of radium is probably because of the differences in the chemical and physical properties of the different cultivating areas.

## Discussion

As scant evidence is linked to the radioactivity of foodstuffs in Ramsar, the results found in this paper represent an important complement to the data of natural radioactivity in foodstuffs in HLNRAs. At present, data on the ^226^Ra radioactivity level in foods are scant in Iran and other countries. Only a limited reliable number of reports are available on evaluation of the ^226^Ra activity levels such as UNSCEAR 2000 that contains reference data on ^226^Ra levels contained in foods.

The radium contamination of the sampled fruits and vegetables does not originate from the deposition of radionuclide particles from the atmosphere onto fruits and vegetables, as they had been washed and the non-edible parts removed. In fact, it is an indirect contamination.

As shown in Fig. 2, in this study the ^226^Ra activity concentrations in different foodstuffs compared with the reference values of UNSCEAR appears to be higher in milk, chicken and eggs and less in grain products, root vegetables, fruits and fish products [3].

Table 3 summarizes the ^226^Ra activity concentrations from the different categories of foods in some countries in the world as reported in UNSCEAR [3].

**Table 3:** The ^226^Ra activity concentration (mBq/kg wet weight) in the different categories of foods in some countries

Categories of Foodstuff	Ramsar-Iran(this study)	Reference value of UNSCEAR	North America United States	Asia	Europe
Milk products	4–64	5	5.7	China 6 Japan 12	Italy 3–19 Germany 2–130 Poland 10 Romania 0.9–44 U.K. <0.4–200
Meat products	<MDA-57	15	20	China 41 Japan 36	Germany 30–220 Poland 11–19 Romania 2–30 U.K. 2.6–74
Grain products	28–70	80	7–100	China 17 Japan 14	Germany 20–2900 Poland 80–110 Romania 30–90 U.K. 0.7–5200
Leafy vegetables	10–530	50	56	China 75	Italy 27–44 Germany 6–1150 Poland 37–43 U.K. 2.2–170
Root vegetables and fruits	7–16	30	7–47	China 63 Japan 11	Italy 14–25 Germany 5–9400 Poland 11–215 Romania 9–190 U.K. 9–41
Fish products	18–43	100	30–59	China 39	France 37 Germany100–7400 Poland 28–43 U.K. 8.5–2100

The mean activity concentrations of ^226^Ra in milk products samples ranged from 4 to 64 mBq/kg. The results of this study are higher than the results presented from Italy, Poland and Romania [3].

The mean activity concentrations of ^226^Ra in meat products samples ranged from <MDA- 57 mBq/kg. The results of this study are comparable with the results reported for China (41 mBq/kg) and Japan (36 mBq/kg), Poland (11–19 mBq/kg), Romania (2–30 mBq/kg), U.K. (2.6–74 mBq/kg) and less than the results presented from Germany (30–220 mBq/kg).

In grain products, the mean activity concentrations of ^226^Ra were found from 28 to 70 mBq/Kg. The results of this study are less than the reference level of UNSCEAR [3].

For leafy vegetables, this mean scale was from 10 mBq/Kg to 530 mBq/Kg that is less than the result reported for Germany and is comparable for the other countries which is reported in UNSCEAR [3]. But the results in root vegetables and fruits (7–16) mBq/Kg is less than the results presented for different countries in UNSCEAR [3].

The ^226^Ra concentrations were compared to the UNSCEAR reference values for Fish products groups and it’s appears to be less than reference value and the results presented for Germany. These results indicate that no significant ^226^Ra contamination is present and provide reference values for the foodstuffs in Ramsar.

The ^226^Ra activity concentration found in the present study is compared to a previous study encompassing some foodstuffs of Tehran city in Iran. The results are very close with regard to wheat and eggs, but there is a significant difference in the results of tea [5].

Since the analyzing laboratory was in Tehran, far from Ramsar, there were some limitations in shipping the samples. Furthermore, as the radiochemical analyses were time-consuming and costly the completion of the project took time.

## Conclusions

It is well-known that radionuclides, whether artificial or natural, are present in the environment. People and their foodstuffs are exposed to different types of radiation that originate from Cosmogenic, terrestrial, or natural decay sources, so studies on different transport pathways of environmental radioactivity have been issued in HLNRAs regions. As most types of food contain detectable amounts of radioactivity, which sequentially relocate into the human body through the ingestion pathway, radioactivity amounts in the daily consumed food products collected from Ramsar markets were therefore determined. This report could be considered as the first systematic study on ^226^Ra contents in foodstuff, as the comprehensive study included different samples from major food groups.

Measurements of ^226^Ra contents in food samples were studied using an alpha-spectrometry system. The results indicate that the existence of ^226^Ra in a variety of amounts, depending on the location of the food cultivation, proving the fact that residents could be exposed daily by food consumption, which include foodstuff from local and markets. To compare the results with UNSCEAR reference values, the ^226^Ra activity concentrations concluded from the results appear to be higher in milk, chicken and eggs and less in grain products, root vegetables, fruits and fish products but these results indicate that no significant ^226^Ra contamination is present in market foodstuffs.

Of the total daily dietary ^226^Ra exposure from market consuming foodstuffs for adults in Ramsar, the largest percentage was from wheat. The residents consuming wheat and manufacturing wheat products such as bread, pasta, porridge, crackers, biscuits, pancakes, pies, pastries, cakes, cookies, muffins, rolls, doughnuts, breakfast cereals and so on may receive an elevated dose in the diet. It is necessary to mention, people could receive different internal effective doses, some fractions of which are related to food samples purchased from markets. It is recommended that, based on ^226^Ra concentration in different food types, habit data, amounts of consumption and its contribution to the daily diet of the people, the results of this study would be used to estimate the internal effective dose of residents as well as its related risk.

The radionuclide targeted in the present work did not include the full range of radionuclides that could potentially enter food chains. In particular other natural radionuclides in the uranium and thorium decay series may also be significant in the Ramsar diet. Full dietary modeling on the activity concentrations using Ramsar food intake values will establish the baseline ingestion dose to sections of the Ramsar public.

## Abbreviations

CR: Concentration ratio
EDTA: Ethylene diamine triacetic acid
FAO: Food and agriculture organization (United Nations)
HLNRAs: High level natural radiation areas
HPGe: High-purity germanium
IAEA: International atomic energy agency
ISO: International organization for standardization
LSC: Liquid scintillation procedure counting
MCA: Multi-channel analyzer
MDA: Minimum detectable activity
UNSCEAR: United nations scientific committee on effects of atomic radiation
